# MRNA Transcription, Translation, and Defects in Developmental Cognitive and Behavioral Disorders

**DOI:** 10.3389/fmolb.2020.577710

**Published:** 2020-09-25

**Authors:** Moyra Smith

**Affiliations:** Department of Pediatrics, University of California, Irvine, Irvine, CA, United States

**Keywords:** transcription, translation, enhancers, chromatin, cognition, autism, epilepsy

## Abstract

The growth of expertise in molecular techniques, their application to clinical evaluations, and the establishment of databases with molecular genetic information has led to greater insights into the roles of molecular processes related to gene expression in neurodevelopment and functioning. The goal of this review is to examine new insights into messenger RNA transcription, translation, and cellular protein synthesis and the relevance of genetically determined alterations in these processes in neurodevelopmental, cognitive, and behavioral disorders.

## Triggers of Gene Expression in Brain Neurons

Precise stimuli for gene expression in neurons remain to be elucidated. Early studies revealed that neurotransmitter activation of receptors triggered intracellular signaling. Greenberg and Ziff (1984) provided insights on intracellular signaling processes and release of specific transcription factors including transcription factor (FOS) that passed from the cytoplasm to the nucleus to trigger the expression of early response genes. Yap and Greenberg (2018) emphasized the importance of the epigenome in bringing about behavioral adaptations to specific stimuli.

Abundant evidence of the importance of epigenetic factors and chromatin modifiers in gene expression in brain development and function can be derived from data on the high frequency of mutations in chromatin modifiers in neurodevelopmental, neurocognitive, and neurobehavioral disorders (Tables 1, 2).

**TABLE 1:** Epigenetic and chromatin-related factor defects implicated in neural tube defects (Greene et al., 2011).

DNMT3A	DNA methyltransferase 3 alpha
DNMT3B	DNA methyltransferase 3 beta
HAT	Histone acetyltransferase
HDAC1	Histone deacetylase 1
HDAC2	Histone deacetylase 2
KAT	Lysine acetyltransferases
CECR2	Histone acetyl-lysine reader involved in chromatin remodeling
ARID1B	Component of the SWI/SNF chromatin remodeling complex
SMARCA4	Regulator of chromatin

**TABLE 2:** Epigenetic and chromatin factors with defects in specific cases of autism spectrum disorders (Satterstrom et al., 2020).

ASH1L	ASH1-like histone lysine methyltransferase
CHD8	Chromodomain helicase DNA-binding protein 8
KDM6B	Lysine demethylase 6B, demethylates di- or tri-methylated lysine 27 of histone H3
KDM5B	Lysine-specific histone demethylase
KMT2C	Histone lysine methylation
KMT2E	Lysine methyltransferase E
SETD5	SET domain proteins function as histone methyltransferases
MBD5	Methyl-CpG-binding domain protein 5
MECP2	Methyl-CpG-binding protein 2

## Transcription Initiation, Promoters, and Enhancers

### Promoters, Enhancers, and Interactions

Haberle and Stark (2018) reviewed core promoters and transcription initiation. They also considered promoter core function in light of chromatin architectures and interactions with regulatory elements.

The transcription start site was noted to be embedded in the core promoter, and the core promoter was defined as including sequence approximately 50 bp upstream and 50 bp downstream of the transcription start site.

Sequence elements in the promoter region act as binding sites for transcription factors, including general transcription factors and transcription cofactors, sequence elements for enhancer binding and the promoter region forms an anchoring site for RNA polymerase II.

Specific sequence elements occur in some, but not all, promoters; these included the TATA box and a sequence element defined as the initiator motif. Haberle and Stark (2018) noted that in human, promoter sequences are often associated upstream with CpG dinucleotide elements.

Specific features of promoter-associated chromatin have been defined that are strongly associated with transcription; these include H3K4me3 and H3K27ac. Transcription initiation at core promoters is defined as a stepwise process that includes recruitment to the promoter of general transcription factors TFIID, TFIIA, TFIIB, and RNA polymerase II, followed by recruitment of general transcription factors TFIIE and TFII H.

Benabdallah and Bickmore (2015) and Haberle and Stark (2018) noted that promoters may establish contact with enhancer elements through chromatin looping.

### Enhancers

Karnuta and Scacheri (2018) reviewed enhancers that they defined as regulatory elements essential for spatiotemporal gene expression. Enhancers were reported to be primarily located in non-protein-coding regions of the genome and to be transcription factor recruiting elements. Genomic analyses have revealed that enhancer element sequences were not conserved across species; however, sequences of specific enhancer elements were highly constrained across humans.

Karnuta and Scacheri (2018) noted that active enhancer elements occur in DNAse 1 hypersensitive chromatin and that chromatin associated with active enhancer elements contained monomethylated histone H3 (H3K4me1) and acetylated histone H3 (H3K27ac). Enhancers not in an active state were reported to be in regions rich in H3K27me3.

Specific histone methyl transferases and histone demethylase interact with enhancers. Enhancer activation was considered to be a stepwise process, and active enhancers could be transcribed by RNA polymerase II to yield enhancer RNA (eRNA) transcripts. The eRNA level indicated the degree of activation of the enhancer. The eRNA then interacted with the gene promoter, and this interaction was mediated by cohesin and mediator complexes. Highly active enhancers were sometimes referred to as super-enhancers, and these were often several kilobases in length.

Karnuta and Scacheri (2018) reported that mutations in genes involved in encoding products involved in enhancer modification or enhancer interactions with promoters can lead to specific diseases. These include mutations in genes that impact histone methylation, histone methyltransferase and histone demethylase, and histone acetyltransferase and histone deacetylases. In addition, defects can occur in chromatin remodeling proteins and proteins that mediate the interaction of enhancer elements with RNA polymerase. Specific factors that facilitate enhancer promoter interactions include mediator complex components and LDB1 (Lim domain proteins).

Schoenfelder and Fraser (2019) also reported that specific diseases result from enhancer dysregulation.

In Table 3, enhancer-related functions and defects are presented.

**TABLE 3:** Enhancer functions and defects.

(A) Specific syndromes in which enhancer modification and/or function was impaired.	
Kabuki syndrome	Due to defects in histone methyltransferase or histone demethylase
Rubinstein-Taybi syndrome	Due to defects in histone deacetylase activity
Charge syndrome	Due to defects in CHD7 chromatin remodeler
Coffin Siris syndrome	Due to defects in chromatin remodelers ARID1A/B, SMARCA/B
Potocki Schaffer	Due to defects in PHF21A component of histone deacetylase
Cornelia de Lange syndrome	Defects in association of enhancer and RNA polymerase II

**(B) Specific proteins with defects that lead to enhancer dysregulation**	

NIPBL	Cohesin loading protein defects
SMC1	Member of the cohesin multiprotein complex
SMC4	Structural maintenance of chromosomes
BRD4	Bromodomain-containing protein involved in chromatin interactions

### Enhancer Variants Documented in Genome-Wide Association Studies

Karnuta and Scacheri (2018) noted that genome-wide association studies have sometimes revealed an association of variants in specific enhancer elements and susceptibility to certain common diseases, including heart disease, type II diabetes, and multiple sclerosis.

It is important to note that enhancers may also function in trans relative to the genes they regulate. Enhancers that function in cis relative to the genes they regulate may employ chromatin looping in gene interactions.

Schoenfelder and Fraser (2019) reviewed enhancer promoter contacts in the control of gene expression. They defined enhancers as regulatory elements that controlled cell type-specific spatiotemporal gene expression. They considered specific challenges in determining mechanisms that underlie promoter enhancer interactions. In addition to evidence of enhancer element promoter interaction through chromatin looping, there was also evidence for non-contact enhancer promoter interactions. Schoenfelder and Fraser (2019) considered details on the expression of the Sonic Hedgehog (Shh) locus to be a prototype of enhancer interactions. Studies in the mouse revealed that Shh gene expression is impacted by an array of tissue-specific enhancers.

Fishilevich et al. (2017) developed a database that documented human enhancers and their target genes. They noted that in addition to contact of enhancers with specific promoters through chromatin looping, enhancer elements can be transcribed to generate eRNAs. Interestingly, eRNAs were shown to frequently be co-expressed with the genes they regulate.

### Long Intergenic Non-coding RNAs

There is now evidence that a large proportion of the human genome is transcribed, and increasingly, long non-coding RNAs are identified. Ransohoff et al. (2018) distinguished between long non-coding RNA that may have some overlap with coding segments in the genome and long intergenic non-coding (linc) RNAs defined as non-coding RNAs greater than 200 nucleotides in length with no overlap with protein coding segments.

Linc RNAs were reported to be primarily present in the nucleus, and their functions were reported to be regulating chromatin topology and formation of scaffolds for binding of proteins. Linc RNAs were reported to stabilize chromatin and to also play roles in chromatin looping.

Gil and Ulitsky (2020) distinguished between cis acting and transacting long non-coding RNAs and noted that they altered the expression of target gene and played roles in tuning spatial and temporal gene expression. They also noted that some long non-coding RNAs included enhancer elements.

Specific architectural proteins including CTCF CCCTC-binding factor, YY1 (transcription factor), and cohesin complex subunits were reported to be anchors of chromatin loops.

## Chromatin Loops

1. Chromatin loops were shown to be important features of the vertebrate genome and to serve to bring enhancers into contact with promoters. The importance of this process was demonstrated in erythroid cells, and a specific upstream locus control region was brought into contact with the beta globin gene locus (Crossley and Orkin, 1993; Tolhuis et al., 2002). Important factors in loop formation included cohesin, CTCF, and protein that included YY1 transcription factor and the zinc finger protein ZNF143. Mediator protein found to be present as chromatin loops brought enhancers into contact with promoters. Cohesin is frequently illustrated as a ring complex and as the architectural unit that surrounds the looped chromatin. The NIPBL protein was reported to load cohesin onto chromatin (Ball et al., 2014).

Core elements of the cohesin complex are presented in Table 4.

**TABLE 4:** Components of the cohesin complex.

SMC1 (SMC1A)	Structural maintenance of chromosome 1A
SMC3	Structural maintenance of chromosome 3
RAD21 (SCC1)	Nuclear phosphoprotein
NIPBL (SCC2)	Cohesin loading factor
SCC4 (MAU2)	Sister chromatid cohesion factor
STAG2	Stromal antigen 2, reported as cohesin complex component

Mutations in SMC1, SMC3, RAD21, and NIPBL have been reported to lead to Cornelia de Lange syndrome associated with developmental defects in a number of systems (Dowsett et al., 2019).

### Topologically Associated Domains

Topologically associated domains (TADS) were defined as sub-megabase-sized domains in which preferential intra-domain interactions occurred, indicating that DNA elements within the TADS interact (Lupiáñez et al., 2016). Genomic alterations that disrupt TADS potentially have a significant impact on enhancer promoter interactions (Hansen et al., 2018).

Specific architectural proteins including CTCF CCCTC-binding factor, YY1 (transcription factor), and cohesin complex subunits were reported to be anchors of chromatin loops.

Developmental defects can also result from structural chromosome changes that disrupt TADS.

There is evidence that chromatin loop formation organized by cohesin and CCCTC binding factor (CTCF) plays important roles in the regulation of gene transcription and in the organization of the 3-dimensional genome. Chromosomes, chromatin, chromatin regulators, and architectural proteins were reported to be involved in the formation of the 3-dimensional genome that has been shown to play an important role in gene expression.

Nasmyth and Haering (2009) reported that cohesin was a key factor in mediating the cohesion of sister chromatids of chromosomes (Table 4). Newkirk et al. (2017) reported that there is growing evidence of the importance of cohesin as a regulator of gene expression.

Other factors that promote the cohesion of sister chromatids include ESCO 1, ESCO2 establishment of sister chromatid cohesion N-acetyltransferase 1 and 2, PDS5 cohesin associated factor B, and Sororin also known as CDCA5 cell cycle division-associated 5.

### Divergent Transcription Directionality

Lacadie et al. (2016) reported that analysis of RNA has revealed the presence of stable RNA generated in a forward direction from a specific promoter and also other unstable transcripts generated from that same promoter in a reverse direction. Directionality of transcription was reported to be influenced by epigenetic factors and chromatin state.

## MRNA Transcription Termination, Polyadenylation, and Alternate Sites

Tian and Manley (2017) reviewed transcription termination, mRNA processing and modification. MRNA transcripts generated by RNA polymerase II undergo maturation through endolytic cleavage of the 3′-untranslated region (UTR) and synthesis of polyadenylation through activity of Poly A polymerase. The site in the mRNA transcript at which cleavage and polyadenylation occur have defined upstream nucleotide elements particularly a hexamer AAUAAA or AUUAAA and downstream of this hexamer U and G rich elements occur GUGU. Frequently a gene manifests more than one potential polyadenylation site. The length of the 3′-UTR of the transcript differs dependent on which polyadenylation site is used.

Tian and Manley (2017) reported that in vertebrates the polyadenylation process requires activity of a core complex of at least 20 proteins referred to as cleavage polyadenylation specific factors. Other proteins involved in polyadenylation include Symplekin, Retinoblastoma binding protein (RBBP6), polyA polymerase (PAP) and CTD defined as an RNA polymerase regulatory protein with a regulatory carboxyterminal domain. The length of the polyA sequence is also dependent upon nuclear PolyA binding proteins PABPN1.

The length of the 3′UTR region upstream of the polyadenylation site utilized influences availability of sites for binding of microRNAs and for binding of other RNA-binding proteins.

Tian and Manley (2017) noted that the length of the 3′UTR region in a processed mRNA influences its ultimate subcellular location. One interesting examples occurred in neurons where the short isoform of the processed mRNA for the neurotrophic factor BDNF was found to occur in the neuronal cell body and the long form of processed BDNF mRNA occurred in dendrites.

Tian and Manley (2017) also noted that alternative polyadenylation site usage and length of the 3′-UTRs of mRNAs of specific genes correlated to some extent with cell differentiation and proliferation. Interestingly, this was proposed to be associated with the level of resistance of the mRNA to binding with microRNAs. Binding of microRNAs to mRNAs occurred preferably in MRNA transcripts with longer 3’-UTRs, and MRNA degradation occurred more rapidly when microRNAs could bind to the mRNA transcript.

Factors in the polyadenylation complex were also noted to be more abundant in proliferating stem cells and in pluripotent stem cells.

Use of proximal polyadenylation sites was also noted when neuronal cells were activated by membrane depolarization. Activation of mammalian target of rapamycin (mTOR) signaling was also reported to lead to the generation of transcripts with shorter 3′-UTRs.

Tian and Manley (2017) also noted that polyadenylation sites sometimes occur in an intron upstream of the last protein coding exon, leading to the generation of a skipped last exon. The skipping of a terminal exon was noted to sometimes modify the function of the protein product of the gene.

### Transcription Factor Defects That Occur in Some Cases of Autism

Voineagu et al. (2011) and Satterstrom et al. (2020) reported finding defect transcription factors in specific autism cases; these are presented in Table 5.

**TABLE 5:** Transcription factors defective in some cases of autism.

FOXP1	Forkhead box P1, transcription factor
MED13L	Mediator complex subunit 13, transcriptional co-activator
POGZ	POGZ
TBR11	T-box brain transcription factor 1
TCL7L2	Transcription factor 7-like 2
TRIP2 (MED1)	Mediator complex subunit 1, activation of gene transcription
SIN3A	Transcription regulator family member A
FOXP2	Subfamily P of the forkhead box (FOX) transcription factor family

## MRNA Splicing and the Spliceosome

Core splicing elements were noted to include 5′ and 3′ splice sites at exon intron junctions, branch points, and polypyrimidine tracts within introns. In addition, splicing is influenced by enhancers and silencers, nucleotide elements that can have trans effects in splicing machinery. Splicing is also dependent on appropriate function of components of the spliceosome.

Genome-wide association studies in autism demonstrated an autism spectrum disorder-associated variant in PTBP2 polypyrimidine tract binding protein 2 that binds to intronic polypyrimidine clusters in pre-mRNA molecules and is implicated in controlling the assembly of other splicing-regulatory proteins.

The composition of the highly dynamic spliceosome was reviewed by Will and Lührmann (2011). They distinguished between U12 and U2 spliceosomes. Their review concentrated on U2 spliceosomes. They noted that the splicing branch site is usually located 18–40 nucleotides upstream from the 3′ splice site and is followed by polypyrimidine sequences. They also noted the presence of splice enhancer and splice repressor elements. Their review concentrated primarily on transacting factors that promoted interactions of the pre-mRNA with the spliceosome.

The spliceosome is described as a multi-megadalton ribonucleoprotein complex. It contains small nuclear ribonucleoproteins (snRNPs) and many other proteins. Core elements recognized by the spliceosome include the 5′ donor site GU dinucleotides, the branch point followed by the polypyrimidine tract and subsequently the AG dinucleotide at 3′ splice acceptor site.

Charenton et al. (2019) reported that the prespliceosome includes U1 and U2 snRNPs that bind to the pre-mRNA 5′ splice site and also to sequences at the splice branch point. This complex then associates with U4, U6, and U5 small nucleoproteins to form the precatalytic spliceosome.

Alternative splicing was reported to be highly regulated and to be responsive to developmental and environmental stimuli (Montes et al., 2019). Alternative splicing is impacted by splice regulatory proteins reported to be rich in serine arginine residues; these can include heterogeneous nuclear ribonucleoproteins (hnRNPs). These are defined as proteins that interact with pre-mRNAs and influence processing including splicing. Polypyrimidine tract binding proteins, e.g., PTBP1, also play important roles in alternative splicing.

The *SNRPN* gene located in chromosome 15q11.2 in the region involved in Prader–Willi syndrome is reported to encode a component of the snRNP complex, expressed from the paternally derived chromosome. It functions in pre-mRNA processing and may contribute to tissue-specific alternative splicing.

## Microexons and Their Inclusion in Transcripts of Genes Expressed in Neurons

Irimia et al. (2014) described a specific program of alternative splicing that led to the inclusion of microexons. Particularly significant was the fact that this program was misregulated in cases of autism. Key to this process of microexon inclusion was the binding of a neuronal splicing regulator factor to intronic enhancer motifs.

Irimia et al. (2014) reported that microexon inclusion particularly impacted transcripts of genes with the following locations or functions: cytoskeleton structure, GTPase regulation, synapses, neurogenesis, vesicle trafficking, calcium signaling, protein kinases. Microexon inclusion was reported to be particularly important during neurogenesis (Table 6).

**TABLE 6:** Genes with misregulated microexon inclusion in autism or intellectual disabilities.

DNTT	DNA nucleotidylexotransferase (AU)
ANK2	ANKYRIN2 links membrane proteins to underlying cytoskeleton (AU)
ROBO1	Roundabout guidance receptor 1 (AU)
SHANK2	SH3 and multiple ankyrin repeat domains 2 (AU)
APBB1	Amyloid beta precursor protein-binding family B member 1 (ID)
TRAPPC9	Trafficking protein particle complex 9 (ID)
RAB3GAP1	RAB3 GTPase activating protein catalytic subunit 1 (ID)

Irimia et al. (2014) reported that neuronal microexon inclusion was misregulated in autism. They reported that extensive transcriptomic studies on brain samples from autistic subjects revealed evidence of altered gene expression in 2,519 genes, and 26.9% of these genes had transcripts that included microexons. The genes with misregulated microexon inclusion in autism were particularly involved in synaptic biology and axonogenesis.

### Neuronal Splice Regulator nSR100/SRRM4 and Its Misregulation

Calarco et al. (2009) described a serine arginine repeat protein that functions as a splice regulator that regulated inclusion of microexons in transcripts of certain brain genes. Calarco et al. (2009) reported that nSR100, also known as SRRM4, directed microexon inclusion and was particularly important in genes involved in neuronal development and function. Specific enhancer elements were reported to occur in genomic DNA between polypyrimidine tract and splice acceptor sites, and these elements bound nSR100 and activated exon inclusion. The transcripts of nSR0100 are particularly abundant in brains; low levels of expression were also reported to occur in the adrenal gland.

Numerous studies in humans with autism have revealed altered neuronal excitation–inhibition ratios resulting from altered neuronal activity and synaptic function. Many different gene defects have been reported to be associated with autism manifestations. In addition, microarray analyses and RNA sequence analyses have revealed altered patterns of gene expression. Misregulation and reduced transcription of the RBFOX family of RNA-binding splice regulators were reported by Voineagu et al. (2011) and Weyn-Vanhentenryck et al. (2018). Irimia et al. (2014) reported altered neuronal microexon splicing in autism.

Irimia et al. (2014) reported that microexons include 3–27 nucleotides and were reported to encode protein fragments that impacted protein–protein interactions. Inclusion of microexons was reported to be controlled by nrS100/SRRM4. Importantly, levels of proteins that are produced from mRNAs with microexon inclusion were reported to be decreased in brains from autistic subjects. Alternative splicing was reported to be regulated by neuronal activity.

Quesnel-Vallières et al. (2016) reported results of studies on mice heterozygous for mutations leading to reduced expression of nSR100/SRRM4, and they analyzed the effect on neuronal activity. They also noted reports on studies on neurons derived from pluripotent stem cells generated from patients with autism that revealed changes in microexon inclusion in neurons in response to nSR100 mutations.

The specific nSR100 defects investigated by Quesnel-Vallières et al. (2016) included deletions of exons 7 and 8 in the encoding gene. Mutant mice were reported to have normal locomotion and normal hearing but abnormal responses to light stimuli and increased startle response. The mice with these mutations were also reported to be averse to novel environment changes and averse to exposure to strange mice. Cortical neurons were reported to have more excitatory glutamatergic neurons and fewer inhibitory Gaba-ergic neurons. However, spines of glutamatergic neurons were noted to be thinner, perhaps as a compensatory mechanism. Analyses revealed increased skipping of microexons in neuronal genes.

Quesnel-Vallières et al. (2016) documented the ontology of neuronally expressed genes that have microexons included in transcripts. Particularly abundant were genes that encoded products involved in vesicle-mediated transport, actin filament organization in cytoskeleton, microtubule function, and GTPase activity and proteins present in Golgi and in cytoskeleton.

The activity of nSR100 was shown to be important in the inclusion of microexons in the transcripts of EIF4G component of the transcription initiation complex, in MEF2C transcription factor transcripts, and in transcripts shown to be aberrant in autism including ANK2, NBEA (neurobeachin), NRXN2 (neurexin 2), and in SHANK 1 (SH3 and multiple ankyrin repeat domains 1).

Importantly, they postulated that nSR100.SRRM4 could potentially be a target for diagnostics and therapeutic intervention in autism.

Parras et al. (2018) reported decreased inclusion of a specific microexon in CPEB4, a polyadenylation element binding protein, in cases of autism. They reported that decreased inclusion of this microexon reduced poly A tail length and expression of the protein product of the transcript.

### Alternative Splicing in Neurodevelopment

Grabowski (2011), Raj and Blencowe (2015), and Weyn-Vanhentenryck et al. (2018) reported that alternative splicing is a key feature in neurodevelopment. Weyn-Vanhentenryck et al. (2018) analyzed temporal regulation of alternative splicing in mouse brain and on purified neuronal subtypes. They determined that there were early-switch and late-switch exons that correlated with neuronal maturation stages.

Early-switch exons were reported to be enriched in the transcripts of genes that encoded ion channels and in genes that encoded products related to transmembrane transport and synaptic transmission. Late-switch exons were enriched in transcripts of genes involved in cytoskeletal remodeling and in the formation of synaptic connections.

In mouse brain, Weyn-Vanhentenryck et al. (2018), also identified distinct RNA-binding proteins involved in the regulation of splicing switches. These gene products included Rbfox, Nova (alternative splicing regulator), Mbnl1 (muscleblind like splicing factor), and Ptbp1 (polypyrimidine tract-binding protein). Rbfox and Nova were reported to promote mature splicing patterns, while Ptbp1 was reported to suppress mature splicing patterns.

Posttranscriptional mechanisms, including alternative splicing, in neural developments, maturation, and plasticity, were reviewed by Furlanis and Scheiffele (2018). In their review, they particularly concentrated on roles of nucleotide codes in RNA transcripts and on RNA-binding proteins in alternative splicing regulation. They noted that alternative splicing of mRNA alters transcript stability, localization, and cell type specificity. They note further that additional studies are required to establish protein changes and possible functional changes brought about by the alternate splicing of specific gene transcripts.

Information on the functional roles of alternative splicing in particular genes has sometimes been derived from information on pathology and disease manifestation that result from splicing mutations in specific genes.

Insights into splice regulation have also been obtained through studies on specific nucleotide elements that interact with RNA-binding proteins and the precise positions of these binding elements relative to alternate exons.

Furlanis and Scheiffele (2018) noted that the Nova splice regulator binds to a YCAY (pyrimidine cytosine adenine pyrimidine) sequence motive positioned approximately 200 bp downstream of an alternate exon, and this binding promotes inclusion of the alternate exon. Location of the YCAY sequence element upstream of an alternate exon was noted to impair inclusion of the exon.

They noted that the presence of cell type heterogeneity in tissues complicates alternative splicing studies. In recent years, transcription studies are increasingly carried out on single cell or on samples with a uniform cell population.

Another important factor to consider is that specific RNA-binding proteins require modifications, and changes in these modifications can impact their binding properties.

Furlanis and Scheiffele (2018) reviewed the neuron splice regulators Nova1/s and nPTB (PTBP2)/RNA-binding proteins referred to as KH RNA-binding domain containing signal transduction associated in humans KHDRBS, KHDRBS2.

They considered whether transcript splicing patterns impacted circuit wiring in the brain and noted that there was evidence that alternate splicing of specific transcripts impacts axonal guidance and synapse formation. This information was derived primarily from studies in *Drosophila* and studies of the transmembrane receptor Dscam1. Evidence was obtained indicating that extensive alternative splicing of Dscam 1 occurred, leading to the generation of products with different numbers of Ig domain inclusions. There was evidence that in dendrite arbor formation, neurites expressing the same number of repeat containing Dscam1 molecules formed connections with each other while dendrites each expressing different Dscam1 isoforms that differed in the number of Ig repeat domain repulse each other and failed to connect. The Dscam1 gene in *Drosophila* is the paralog of a human gene DSCAM that is impacted in Down syndrome.

The DCC guidance receptor, also known as DCC netrin receptor, was shown to undergo alternate splicing events in mice, and the specific isoform expressed impacted axon guidance and interaction.

Furlanis and Scheiffele (2018) cited evidence that voltage-gated calcium channel transcripts have multiple splice variants and that the presence of specific variants impacts cell type specificity of the specific calcium channel. These channels play key roles in neurotransmitter release in synapses.

Transcripts of neurexin, the synaptic adhesion molecule, were reported to exhibit significant differences based on splice site usage differences.

Transcripts of KCNMA1, the potassium calcium-activated channel, were also reported to exhibit splicing differences, leading to protein products that differentially influence neuronal excitability.

Furlanis and Scheiffele (2018) noted that there is evidence that in cortical neurons, intron-containing transcripts derived from a number of genes occur in the nucleus and that these transcripts undergo splicing in response to specific signals.

### Intronic Variants Leading to Aberrant Splicing

Mutations in genes that encode sodium ion channels are important causes of epilepsy. Mutations in the SCNA genes are reported to be associated with Dravet syndrome, an autosomal dominant disorder associated with encephalopathy, epilepsy, and developmental delay.

Carvill et al. (2018) described seven likely pathogenic SCN1A variants in patients with epilepsy where the variants occurred outside the protein codon region of the genes. Five of the variants occurred in introns and led to the inclusion of an abnormal exon. They described the abnormal exon as a poison exon, since it led to a decreased production of active SNC1A protein. These five intron variants occurred in intron 20. This intron is reported to have a highly conserved sequence in mammals. In two of the five epilepsy cases, the variant involved a short deletion of nucleotides, and in three cases, the intronic variant was a single nucleotide substitution. Four of the five patients with this abnormal intron had Dravet syndrome, and one individual had febrile seizures. In two of the cases, the variant occurred in other members of the same family who had febrile seizures. The five intron variants were not present in population data.

### Pathogenic Repeat Sequence Expansions in Introns

Ishiura et al. (2018) reported abnormal nucleotide repeat expansion in intron 4 of the SAMD12 sterile alpha motif-encoding gene that is normally abundantly expressed in the brain of individuals with familial myoclonic epilepsy. The repeat expansion involved TTTCA and TTTTA repeats. They noted that repeat expansions of TTTTA and TTCA were noted in two different genes TNRC6A and RAPGEF2 in cases of familial myoclonic epilepsy. TNRC6A encodes a protein that functions in posttranscriptional gene silencing through the RNA interference (RNAi) and microRNA pathways. RAPGEF2 Rap guanine nucleotide exchange factor 2 functions in signal transduction.

Corbett et al. (2019) reported intronic ATTTC repeat expansions in a specific gene STARD7 in cases of adult myoclonic epilepsy. The ATTC repeat expansion in the first intron of STARD7 was identified in 150 individuals in 16 different pedigrees. STARD7 is defined as StAR-related lipid transfer domain containing 7.

Florian et al. (2019) reported that intronic repeat expansion was reported to be among the most important causes of familial adult myoclonic epilepsy in Asia. They reported cases of familial adult myoclonic epilepsy with intronic repeat expansion in the gene that encodes MARCHF6 membrane associated ring-CH-type finger 6, a membrane-associated E3 ubiquitin ligase. They reported that the degree of repeat expansion was variable 3.3–14 kb. The large repeats sometimes predisposed to genomic instability of the 5p15.2 region in which they were located.

## Rare Variant Interpretations in Light of Transcript Expression

Cummings et al. (2020) stressed the importance of cell and tissue transcription differences into account when interpreting the pathological significance of a specific genomic variant identified in an individual with a specific disease.

Through studies in autism and developmental disabilities, they provided evidence that putative loss of function variants in weakly expressed exons in neuronal cell types has the same level of significance as synonymous variants.

They noted that no information tools currently exist for incorporation of transcript analysis into variant interpretation.

Cummings et al. (2020) developed a transcript level annotation tool designated proportion expressed across transcripts (pext). They reported that the metric they developed was useful in filtering isoforms with low levels of expressivity. It was, however, less useful in filtering variants with intermediate levels of expression.

## MRNA Degradation

MRNA degradation processes include mechanisms involved in degradation of normal MRNA and also additional processes involved in degradation of mutant MRNA. Degradation processes include endonucleolytic cleavage, exonucleolytic cleavage, de-adenylation, and exosome activity. Particular processes are involved in the degradation of mRNA forms with premature termination codons.

Exosomes are involved in trimming and degradation of mRNAs. The presence of AU-rich elements in the 3′-UTR of mRNA facilitates binding to exosomes. Müller et al. (2015) drew attention to multiprotein exosome complexes involved in mRNA degradation. Exosome activity also requires the presence of specific cofactors. They presented examples of mutations in the exosome component that impact mRNA degradation.

Importantly, mutations in specific exosome component EXOSC3 were reported to be associated with the neurodevelopmental disorder pontocerebellar hypoplasia. Mutations in the exosome component EXOSC8 was associated with a disorder in children in which cerebellar hypoplasia, hypomyelination, and spinal muscular atrophy occurred.

There is evidence that exosome activity occurs in the nucleus and in the cytoplasm. MRNA to be degraded is reported to be guided to the exosome in the nucleus by a complex referred to as the TRAMP complex. In the cytoplasm, the SKI complex guides MRNA for degradation to the exosome. The MTR4 (MTREX) helicase complex was reported to be important for mRNA degradation in humans.

Müller et al. (2015) emphasized that mutations in the exosome complex functions impacted motor neurons, Purkinje cells, and oligodendroglia. They noted that it was not yet clear why defective exosome function primarily impaired brain and neuronal cells.

### Control of MRNA Levels and Processing

Müller et al. (2015) reported that precise control on MRNA levels and MRNA processing is important in neurons. Control includes levels of transcription and also levels of degradation. Factors that are involved in control include mRNA-binding proteins, long non-coding RNAs, and microRNA activity.

Long non-coding RNAs were postulated by Roberts et al. (2014) to play roles in numerous processes related to gene expression including transcription regulation, epigenetic alterations, and modulation of RNA splicing.

### MRNA Passage From Nucleus to Cytoplasm

MRNA passes from nucleus to cytoplasm through nuclear pore complexes. This complex is composed of multiple proteins including nucleoporins, and energy for transport through nuclear pores is dependent on guanine triphosphate. Exportin proteins also facilitate transport out of the nucleus. In recent years, there are reports of nucleoporin defects in specific neurodevelopmental disorders. RANBP2 is a large protein that localizes to the nuclear pore complex. Defects in RANBP2 have been reported to predispose to familial encephalopathy (Nofrini et al., 2016). Fichtman et al. (2019) reported an association of pathogenic mutations in nucleoporin NUP124 in encephalopathy.

## Translation Processes

Cooper (2000) reviewed protein translation processes in eukaryotes and identified factors involved in each of the three stages of translation, initiation, elongation, and termination.

Gabut et al. (2020) noted that early steps in translation involve bringing the 40s ribosome subunit into contact with the 5′mRNA region through activity of the eIF4 complex. Merging occurs between eIF4E, eIF4G, and eIF4A that together form the eIF4 complex. The eIF4complex merges with the preinitiation complex (PIC).

The preinitiation complex refers to a complex that includes guanosine triphosphate (GTP) bound to eIF2 and methionyl initiator tRNA, eIF3, eIF5, eIF1, eIF1A 40S ribosome subunit. The PIC complex was reported to scan mRNA until the AUG initiation codon was encountered. Subsequently, hydrolysis of the eIF2-bound GTP occurs, leading to the release of many of the bound initiation factors. This facilitates recruitment of GTP-bound eIF5B and the 80S binding, and the remaining bound eIF factors are released. The aminoacyl-tRNA-binding site on ribosomes is exposed, allowing translation extension to occur.

Gabut et al. (2020) stressed that cellular protein synthesis is an energy-intensive process and requires adequate functioning of the mTOR and mitogen-activated protein (MAP) kinase pathways.

Another important factor involved in controlling the degree of cellular protein synthesis is the integrated stress response (Tahmasebi et al., 2018).

Specific translation initiation complex subunits that recognize the 3-end of the mRNA include eif4G and eif4E. Cooper (2000) noted that this indicates the importance of the polyadenylation in the translation process.

Additional initiation factors that serve to bring the mRNA to the ribosome include eiF4E, eiF4G, eiF4A, and eif4b, and eiF4G was reported to also interact with eiF3.

Following this binding, the 40S ribosome subunit with bound eif5 and initiator methionyl tRNA scans the mRNA for the AUG initiator codon. Hydrolysis of GTP on eif2 occurs through the activity of eiF5; the eiF2 is then released, and the 60s subunit of the ribosome joins the 40s ribosomal subunit and translation can then begin.

### Aberrant Translation Initiation

O’Neill et al. (2001) described a large kindred in which several members had X-linked adrenoleukodystrophy and adrenomyelomyopathy due to deletions of the translation initiation codon and utilization of a downstream ATG codon in the ABCD1 gene ATP-binding cassette subfamily D member 1 that encodes a peroxisomal protein. Use of the downstream initiation codon leads to the generation of an aberrant shortened protein. The short protein was associated with impaired function and impaired ability to reduce very-long-chain fatty acids. The excess long-chain fatty acid was reported to accumulate in neural white matter.

## Defects in Translation Initiation Processes in Neurodevelopmental Disorders

In 2012, Borck et al. (2012) identified a translation defect in a specific syndrome with the acronym MEHMO characterized by cognitive impairments, epilepsy, hypogonadism, microcephaly, and obesity. They determined impaired function of the GTP translational initial factor eiF2 and initiator methionyl-tRNA ternary complex that binds to the 40S ribosome subunit and scan mRNA transcripts to select the AUG translation initiation site. An X-linked gene EIFS3 was reported to encode the gamma subunit of eIF2 and was found to have a mutation individual with the MEHMO syndrome. In 2019, Young-Baird et al. (2019) reported identification of a pathogenic EIFS3F mutation in individuals in a different family.

### Non-ATG Translation

Microsatellite nucleotide repeat expansions were reported to be associated with more than 49 different diseases that impact the central and peripheral nervous systems. There is growing evidence that the repeat sequence regions can be transcribed and translated, giving rise to aberrant peptides and proteins. Cleary et al. (2018) reviewed the form of translation that derived from repeat sequence transcripts. This form of translation is known as repeat non-ATG translation or RAN translation.

Cleary et al. (2018) noted that normal translation involves the stepwise activity of multiple proteins. Key steps include recognition of the 5-methyl 7 guanine cap on MRNA followed by recruitment of the 43S preinitiation complex and 5′-UTR scanning until the AUG initiation codon was recognized and paired with metTRNA. The pairing then facilitates, followed by GTP hydrolysis and 60S ribosomal subunit binding, release of many of the preinitiation complex factors, and translation elongation can begin.

The first RAN translation process was recognized in spin cerebellar ataxia cases associated with a CAG repeat expansion. Cleary et al. (2018) noted that, by 2018, RAN-derived proteins had been identified in eight different neurodegenerative disorders associated with nucleotide repeat expansions.

There is some evidence that aberrant dipeptides and short proteins derived from aberrant translation can accumulate in inclusions.

## Translation Elongation and Aminoacyl TRNAs

Translation involves aminoacyl tRNAs being escorted to ribosomes by the GTP-bound elongation factors, e.g., eEf1, eEf1a. On recognition of the cognate nucleotide codon in the mRNA, the specific aminoacyl-tRNA interacts with the ribosome and polypeptide, and the GTP on the elongation factor is hydrolyzed. The elongation process involves movement of the ribosomes along three nucleotides of the mRNA with each addition of an amino acid. Elongation proceeds until a termination codon is encountered in the mRNA transcript.

## Aminoacyl tRNA Synthetases

Antonellis and Green (2008) reported roles of aminoacyl tRNA synthetases in genetic diseases. Aminoacyl tRNA synthetases and their deficiencies were reviewed by Fuchs et al. (2019). Aminoacyl tRNA synthetases are required to correctly couple amino acids to their cognate tRNAs, and specific tRNAs are required to decode specific nucleotide triplets in the mRNA.

Each aminoacyl-tRNA synthetase couples a specific amino acid. Fuchs et al. (2019) emphasized that protein synthesis occurs both in the cytoplasm and in the mitochondria and that some genes encode tRNA synthetases that are active only in mitochondria or in the cytoplasm; other aminoacyl tRNA synthetases function in both mitochondria and cytosol. Note, as presented in Table 7, that the aminoacyl-tRNA synthetases GARS and KARS are bifunctional and act in cytosol and mitochondria.

**TABLE 7:** tRNA synthetase and chromosome locations of encoding genes.

AARS1	Alanyl-tRNA synthetase 1, 16q22.1
AARS2	Alanyl-tRNA synthetase 1, mitochondrial, 6p21.1
DARS1	Aspartyl-tRNA synthetase 1, 2q21.3
DARS2	Aspartyl-tRNA synthetase 2, mitochondrial, 1q25.1
GARS1	Glycyl-tRNA synthetase 1, 7p14.3
HARS1	Histidyl-tRNA synthetase 1, 5q31.3
HARS2	Histidyl-tRNA synthetase 2, 5q31.3, mitochondrial
IARS1	Isoleucyl-tRNA synthetase 1, 9q22.31
IARS2	Isoleucyl-tRNA synthetase 2, mitochondrial, 1q41
KARS1	Lysyl-tRNA synthetase 1, 16q23.1
LARS1	Leucyl-tRNA synthetase 1, 5q32
LARS2	Leucyl-tRNA synthetase 3, mitochondrial, 3p21.32
MARS1	Methionyl-tRNA synthetase 1, 12q13.3
MARS2	Methionyl-tRNA synthetase 2, mitochondrial, 2q33.1
RARS1	Arginyl-tRNA synthetase 1, 5q34
RARS2	Arginyl-tRNA synthetase 2, mitochondrial, 6q15
SARS1	Seryl-tRNA synthetase 1, 1p13.3
SARS2	Seryl-tRNA synthetase 2, mitochondrial, 19q13.2
VARS1	Valyl-tRNA synthetase 1, 6p21.33
VARS2	Valyl-tRNA synthetase 2, mitochondrial, 6p21.33
YARS1	Tyrosyl-tRNA synthetase 1, 1p35.1
YARS2	Tyrosyl-tRNA synthetase 2 mitochondrial, 12p11.2
QARS1	Glutaminyl-tRNA synthetase 1, 3p21.31

## Clinical and Pathological Abnormalities in Patients With Aminoacyl tRNA Function Defects

MRI abnormalities, psychomotor retardation, microcephaly, hypotonia, seizures, and encephalopathy were frequently encountered. Failure to thrive was a commonly encountered early manifestation in children. Other manifestations that were encountered in some patients included liver, bone marrow, and gastrointestinal defects.

Nakayama et al. (2017) reported that specific mutations in AARS, alanyl-TRNA synthetase lead to progressive microcephaly, epilepsy, and spasticity.

## TRNAs

Schaffer et al. (2019) reviewed the roles of tRNAs in neurodevelopment. They reported that there are more than 600 potential TRNA-encoding segments in the human genome. They also noted that brain abnormalities and defects in the peripheral nervous system have been reported to result from abnormalities in tRNA functions (Table 7).

They defined tRNAs as adaptors in the translation process. Multistep maturation processes are required for tRNAs to be functional. In addition, there is evidence that TRNAs have other functions.

In reviewing the tRNA life cycle, Schaffer et al. (2019) noted that tRNA elements in the genome are transcribed by RNA polymerase III that are recruited to TRNA genomic segments by transcription factors TFIIIB and TFIIIC. Polymerase III levels are regulated by MAF1, which must first be phosphorylated to promote RNA polymerase III activity. The tRNA transcripts require modification before they can be utilized.

In the mitochondria, 22 tRNA genes were reported to be active. Products for modifying mitochondrial tRNAs were reported to be encoded in the nucleus and to require transport into mitochondria. Schaffer et al. (2019) noted that more than 100 unique modifications of tRNAs occur.

Following modification, TRNAs occur in the cytoplasm, mitochondria, and endoplasmic reticulum. Specific transporters of TRNAs included exportin 7 encoded by the XPO7 gene.

Twenty different aminoacyl tRNA synthetases occur, each recognizing a specific amino acid.

Schaffer et al. (2019) reported that TRNAs with bound amino acids and with 3′CAA dinucleotides are recognized by the specific eEF2a subunit and bind to the ribosome complex for generation of translation products. The TRNA molecules were reported to be very stable; however, specific catalytic mechanisms are involved in TRNA degradation.

Schaffer et al. (2019) reviewed specific childhood neurological disorders associated with deletions or mutations of specific TRNAs required in the mitochondria. These disorders include Kearns–Sayre syndrome, Leigh syndrome, mitochondrial myopathies, mitochondrial encephalopathies associated with lactic acidosis. Pathologies associated with these disorders were described by Kosmorsky and Johns, 1991.

Childhood neurological disorders associated with impaired biogenesis of nuclear TRNAs are noted to lead to hypomyelinating leukodystrophies and pontocerebellar hypoplasia. Leukodystrophies have also been reported to result from POLR3A or POLR3B defects. POLR3A and POLR3B encode the component of RNA polymerase III that is involved in the transcription of tRNA genes.

Pontocerebellar hypoplasia can result from defects in functions of the products of the TSEN genes including TSEN2, TSEN15, TSEN34, and TSEN54 that encode tRNA splicing endonucleases.

Neurological disorders can also result from defective functions of enzymes that charge tRNAs, namely, TRNA synthetases. Specific syndromic disorders due to defects in aminoacyl-tRNA synthetases include forms of Charcot–Marie–Tooth disease that is characterized by impaired peripheral nerve functions primarily in axons that often first present with abnormalities in the legs. Defects in any one of more than 70 different genes have been reported to lead to this disorder. The PMP2 gene that encodes peripheral myelin protein 22 is the most commonly involved. Defects leading to Charcot–Marie–Tooth disease have been identified in six different aminoacyl-tRNA synthetases, alanyl-t-RNA synthetase 1 (AARS1), methionyl-tRNA synthetase 1 (MARS1), histidyl-tRNA synthetase 1 (HARS1), tyrosyl-tRNA synthetase 1 (YARS), lysyl-tRNA synthetase 1 (KARS), and glycyl-tRNA synthetase 1 (GARS).

Schaffer et al. (2019) noted that another clinicopathologic phenotype with peripheral nerve abnormalities combined with oxidative phosphorylation defects arises due to a defect particularly of aminoacyl-tRNA synthetases that function in the mitochondria. This phenotype can arise due to defects in the following six mitochondrial aminoacyl-TRNA synthetases, glutamyl-tRNA synthetase 2 (EARS2), phenylalanyl-tRNA synthetase 2 (FARS2), threonyl-tRNA synthetase 2 (TARS2), asparaginyl-tRNA synthetase 2 (NARS2), methionyl-tRNA synthetase 2 (MARS2), and alanyl-tRNA synthetase 2 (AARS2).

This combined phenotype can also arise due to a defective function of mitochondrial methionyl-tRNA transformylase (MTFmt) that is required for initiation of translation in the mitochondria.

Schaffer et al. (2019) noted that hypomyelinating leukodystrophies may also result from defects in proteins involved in the charging of TRNAS, aminoacyl-tRNA synthetase complex interacting multifunctional protein 1 (AIMP1) and aminoacyl-tRNA synthetase complex interacting multifunctional protein 2 (AIMP2).

Microcephaly with cerebellar atrophy was reported to result from defects in glutaminyl-tRNA synthetase 1 (QARS1) or defects in seryl-tRNA synthetase 1 (SARS1).

Schuller et al. (2017) noted advances in elucidation of factors that regulate translation termination. The ETF1 factor decodes the termination codons. They noted that although ETF1 constituted in humans the main catalytic factor in translation termination, interaction with other factors was also important. These included ABCE1, an ATP-binding cassette. Proximity to the polyA binding protein (PABP), e.g., PABPC1 (polyA binding protein cytoplasmic), was also important.

Schuller et al. (2017) included an additional process in translation termination, namely, ribosome recycling. The ABCE1 protein was reported to also play roles in releasing ribosomes from mRNA and promoting dissociation into subunits.

Premature termination codons in mRNA elicit a mechanism referred to as nonsense-mediated decay that involves a surveillance complex composed of RENT1 also referred to as UPF1 RNA helicase and ATPase and peptide release factor also known as ERF3A.

## Translation Elongation Complex EIF4F Variants, Mutations, and Roles in Neurodevelopmental Diseases

McLachlan et al. (2019) reviewed the components of the multisubunit protein complex eEF1 that is involved in transcription elongation and plays a key role in protein synthesis. The designated nomenclature of the components of this complex in humans defines genes and protein subunits (Table 8).

**TABLE 8:** Components of the multisubunit protein complex eEF1.

Gene	Protein	Map position
*EEF1A1*	eEF1A1	6q14
*EEF1A2*	eEF1A2	20q13.3
*EEF1B2*	eEF1Balpha	2q33
*EEF1D*	eEF1Bdelta	8q24.3
*EEF1G*	eEF1gamma	11q12.3
*VARS*	Val-RS	6p21.3

The proteins eEF1A1 and eEF1A2 were noted to constitute central components of the complex with eEF1A2 occurring primarily in neurons and eEF1A1 widely present. The EEF1A gene was reported to be mutated in specific cases of autism. McLachlan et al. (2019) noted that the remaining subunits of the complex, eEF1Balpha, eEF1Bgamma, and eEF1Bdelta, and valyl TRNA synthetase were individually reported to be mutated in specific neurodevelopmental defects.

The eEF1A complex was reported to function to deliver aminoacyl-associated tRNAs to the ribosome to generate the polypeptide chain. Energy for this process is provided by GTP.

McLachlan et al. (2019) noted that neurodevelopment is particularly dependent on optimal translation processes. A specific homozygous mutation p.(P333)L in the EEF1A2 gene product was reported in children with global developmental delay, epilepsy, and cardiomyopathy. Nakajima et al. (2015) reported defects in EEF1A2 in patients with intellectual disability, autism, and epilepsy.

Deletion of an exon in the EEF1B2 gene was reported to be present in three siblings with intellectual disability in one family. EEF1D mutations have been reported to be associated with intellectual disability in members of one family. Different VARS gene mutations were reported to occur in two different families with intellectual disability.

McLachlan et al. (2019) noted that the neurodevelopmental defects that result from eEF1 subunit mutations draw attention to the importance of protein homeostasis in neurons. In addition, these studies provided additional evidence that mutations in certain housekeeping genes can lead to neurodevelopmental defects.

## Errors in Translation

Kapur and Ackerman (2018) reviewed errors in mRNA translation and their relevance to neurological diseases. They defined translation errors as including one of the following: incorporation of incorrect amino acids, frame shift errors, readthrough of stop codons, and premature termination of translation.

They noted that advances in molecular techniques have expanded insights into factors involved in translation and its termination. One of these techniques includes ribosome profiling. This technique is designed to determine quantitative measures of protein synthesis. Brar and Weissman (2015) described a method to sequence ribosome-protected mRNA fragments. They reported that the process is based on the fact that about 30 nucleotides of mRNA are found to be protected by the ribosome with each successive movement along the mRNA transcript during the translation process. This protects the ribosome-covered segments from nuclease digestion. The ribosome-protected segments can be isolated, and sequences so-called ribosome footprints can be identified.

A specific aminoacyl-tRNA synthetase must select the correct amino acid, and it must select the correct tRNA from the TRNA pool. Specific editing processes take place. In pretransfer editing, an incorrectly bound amino acid can be removed from the aminoacyl-tRNA synthetase by hydrolysis. Mischarged tRNAs can also be edited. These pre- and posttranslation editing processes require the existence of proofreading mechanisms (Martinis and Boniecki, 2010).

Translation Elongation factor mutations in EEF1A2 were reported in cases of epilepsy, ataxia, intellectual disability, and autistic behavior by Lam et al. (2016). They reported that in these cases, the mutations occurred in sites encoding amino acids that were highly conserved in evolution; EEF1A2 mutations were also reported in patients with intellectual disability and autism by Nakajima et al. (2015).

Translation termination factors were designated eRF, and genes were designated ERF or ETF1. Termination proteins included eRF1, eTF1 (eRF3); a gene encoding etf3 was not found in the human gene database. It is important to note that nomenclature for translation factors remains complex.

### Ribosome Quality Control

A specific process referred to as ribosome quality control (RQC) is initiated when a ribosome stalls during the process of translation. Joazeiro (2019) delineated different factors that lead to ribosome stalling. This can result from abnormal features in the MRNA or abnormalities in the TRNA, and it can also result from defects in ribosomes.

When ribosomes stall, the aberrant or shortened polypeptide chain undergoes proteosomal degradation. Joazeiro (2019) noted that an important factor in this degradation was the ubiquitin ligase, LTN1 listerin 1.

## Joint Interactions of Regulatory and Coding Variants in Determining Penetrance

Castel et al. (2018) reviewed aspects of variable penetrance of disease-causing mutations and the relationship of this to associated regulatory regions. Their studies included analyses of haplotypes of regulatory regions known to impact specific disease-related genes.

Castel et al. (2018) defined variable penetrance as variable severity of the phenotypic manifestations and as the proportion of carriers of a specific disease-causing mutation who manifested pathologic features of the disorder. They also noted that penetrance of specific mutations could be influenced by environmental factors.

Their study focused on analyses of haplotypes of defined regulatory regions that were located in cis to the target gene. They also utilized *in vitro* studies to study the effects of regulatory regions and target genes.

The study was based on the hypothesis that nucleotide variants, the haplotype, in regulatory regions impact penetrance; specific haplotypes reduced penetrance, while other haplotypes were disadvantageous and increased penetrance, while other haplotypes were advantageous and individuals with the advantageous haplotype showed few manifestations of the disease despite the presence of deleterious mutations in the target gene. They also postulated that the deleterious haplotypes were rare in the general population. Patients with manifestations of the disease caused by deleterious mutations in a specific disease would have an overrepresentation of specific regulatory haplotypes relative to the frequency of those haplotypes in the general population.

Among the results of their study was evidence for a regulatory region haplotype that modified the phenotype of Birt–Hogg–Dubé syndrome. This syndrome was reported by Menko et al. (2009) to be due to heterozygous deleterious mutations in the Folliculin gene (FCLN) that maps to chromosome 17p11.2. This syndrome may be associated with cutaneous tumors (folliculomas), renal tumors, colon polyps, and pulmonary cysts leading to pneumothorax.

It is however important to note that there are many other factors that can influence the clinical expressivity and penetrance of a specific deleterious variant. In a review in 2013, Cooper et al. (2013) noted that penetrance and clinical expressivity can be influenced by sex-specific factors, by variable expressivity of alleles at the specific impacted gene locus (especially important to consider in heterozygous conditions), by variants at unlinked modifier loci, by epigenetic factors, and by environmental factors. Also, specific similar clinical manifestations may occur as a result of mutations at more than one gene locus.

## Proteins and Proteomics and Relevance to Functional Studies of Gene Mutations

Yates (2019) reviewed technical advances in proteomics. He noted that challenges remain in uncovering the functions of proteins in cells and that it is important to consider protein–protein interactions and posttranslational modifications.

Posttranslational modifications are often important in regulating protein functions and in determining the locations of specific proteins in specific subcellular organelles. In addition, specific factors influence the correct three-dimensional folding of proteins that may be required for their function.

### Relevance of Protein Subunit Interactions: Sodium Ion Channels and Epilepsy

Wallace et al. (1998) reported that mutations in sodium ion channel gene *SCN1B* played roles in epilepsy. Noebels (2019) reviewed sodium ion channels and their functional changes that play roles in epilepsy. He noted the complexities due to the required interactions of different subunits to form an ion channel, the importance of interactions between different subunits, and complexities due to differences in the expression of specific sodium channels in specific brain regions.

Noebels (2019) noted that the functional sodium channel is a heteromer composed of proteins that include any of the 10 SCN alpha subunits and a beta SCN subunit. Currently, 11 different genes are known to encode alpha subunits of the sodium ion channels in the brain, SCNA1 to SCN11A, and one gene encodes the beta subunit SCN1B expressed in the brain.

Noebels (2019) noted that alterations in splice site use lead to generation of different splice isoforms and that different isoforms may predominantly occur in different cell types.

Sodium ion channel proteins occur in neuronal soma and in dendrites and to have high density in the axon initial segment and in the myelinated node of Ranvier. Sodium ion channels are reported to initiate electrical neuronal action potentials.

Five different genes that encode alpha subunits of sodium ion channels have been reported to have mutations that lead to epilepsy; these include SCN1A, SCN2A, SCN3A, SCN8A, and SCN9A, and epilepsy-inducing mutations have also been reported in SCN1B.

In addition, posttranslational modifications are required for the sodium ion channel functions. The extent to which specific amino acid residues are involved in posttranslational modifications needs to be considered in analyses of the impact of specific mutations on function.

The subunit interactions are also impacted by the degree to which posttranslational modifications impact the subunit interactions and protein functions.

## Author Contributions

MS reviewed literature and generated the manuscript.

## Conflict of Interest

The authors declare that the research was conducted in the absence of any commercial or financial relationships that could be construed as a potential conflict of interest.
